# Total Occlusion of the Infarct-Related Artery in Non-ST-Elevation Myocardial Infarction (NSTEMI)—How Can We Identify These Patients?

**DOI:** 10.3390/medicina57111196

**Published:** 2021-11-03

**Authors:** Irmina Morawska, Rafał Niemiec, Maria Stec, Karolina Wrona, Paweł Bańka, Andrzej Swinarew, Maciej Wybraniec, Katarzyna Mizia-Stec

**Affiliations:** 1 Upper Silesian Medical Centre, Students’ Scientific Society of the First Department of Cardiology, School of Medicine in Katowice, Medical University of Silesia, 40-055 Katowice, Poland; irmina.morawska@gmail.com (I.M.); niemiec.raf@gmail.com (R.N.); mariaannastec@gmail.com (M.S.); 2 Upper Silesian Medical Centre, First Department of Cardiology, School of Medicine in Katowice, Medical University of Silesia, 40-055 Katowice, Poland; karolina.wrona.gcm@gmail.com (K.W.); pawelbanka19@gmail.com (P.B.); maciejwybraniec@gmail.com (M.W.); 3 Faculty of Computer Science and Material Science, Institute of Material Science, University of Silesia in Katowice, 40-055 Katowice, Poland; andrzej.swinarew@us.edu.pl

**Keywords:** non-ST-segment elevation myocardial infarction, total occlusion, infarct-related artery, clinical predictors

## Abstract

*Background and Objectives:* Regardless of the improvement in key recommendations in non-ST-elevation myocardial infarction (NSTEMI), the prevalence of total occlusion (TO) of infarct-related artery (IRA), and the impact of TO of IRA on outcomes in patients with NSTEMI, remain unclear. *Aim:* The study aimed to assess the incidence and predictors of TO of IRA in patients with NSTEMI, and its clinical significance. *Material and Methods:* The study was a single-center retrospective cohort analysis of 399 consecutive patients with NSTEMI (293 male, mean age: 71 ± 10.1 years) undergoing percutaneous coronary intervention. The study population was categorized into patients with TO and non-TO of IRA on coronary angiography. In-hospital and one-year mortality were analyzed. *Results:* TO of IRA in the NSTEMI population occurred in 138 (34.6%) patients. Multivariate analysis identified the following independent predictors of TO of IRA: left ventricular ejection fraction (odds ratio (OR) 0.949, *p* < 0.001); family history of coronary artery disease (CAD) (OR 2.652, *p* < 0.001); and high-density lipoprotein (HDL) level (OR 0.972, *p* = 0.002). In-hospital and one-year mortality were significantly higher in the TO group than the non-TO group (2.8% vs. 1.1%, *p* = 0.007 and 18.1% vs. 6.5%, *p* < 0.001, respectively). The independent predictors of in-hospital mortality were: left ventricular ejection fraction (LVEF) at admission (OR 0.768, *p* = 0.004); and TO of IRA (OR 1.863, *p* = 0.005). *Conclusions:* In the population of patients with NSTEMI, TO of IRA represents a considerably frequent phenomenon, and corresponds with impaired outcomes. Therefore, the utmost caution should be paid to prevent delay of coronary angiography in NSTEMI patients with impaired left ventricular systolic function, metabolic disturbances, and a family history of CAD, who are at increased risk of TO of IRA.

## 1. Introduction

The resting 12-lead ECG is the first-line diagnostic tool in assessing patients with suspected acute coronary syndrome [1,2]. The presence of ST-segment elevation on an ECG is an indication for immediate coronary angiography and revascularization. Total occlusion (TO) of infarct-related artery (IRA) is typical for ST-segment elevation myocardial infarction (STEMI), and is associated with greater extent of necrosis, and worse in-hospital and short-term prognosis [3]. Although the certain time delay of percutaneous coronary intervention (PCI) in non-ST segment elevation myocardial infarction (NSTEMI) is acceptable, clinically, some NSTEMI patients also experience TO of the IRA [4,5,6]. Complex biochemical mechanisms, as well as erosion of the plaque, legitimize these procedures, however, the clinical assessment may be difficult [7,8]. Thus, an early routine invasive approach within 24 h of admission is recommended for NSTEMI, based on high-sensitive cardiac troponin (hs-cTn) measurements, GRACE risk score > 140, and dynamic new, or presumably new, ST-segment changes [1].

Regardless of the novel reassessment of mechanisms of plaque rupture and improvement in NSTEMI early invasive strategy [1], the delay in an invasive approach in patients with TO of IRA seems important.

Previous studies have reported that TO of IRA significantly increased the mortality in patients with NSTEMI [9] and shed light on the stronger predictive value of electrocardiographic findings [1] or clinical presentation on the outcomes in this group of patients. 

The study aimed to analyze the incidence and the clinical predictors of TO of IRA in patients with NSTEMI undergoing percutaneous coronary intervention (PCI), and its clinical significance in a one-year follow-up.

## 2. Materials and Methods

### 2.1. Study Population

The study was a single-center retrospective cohort analysis of 399 consecutive patients with NSTEMI (293 men, mean age 71 ± 10.1 years) undergoing PCI hospitalized in the 1st Chair and Department of Cardiology, the Medical University of Silesia in Katowice between the 1st of January and the 31 December 2019.

All patients were treated according to the 2015 European Society of Cardiology (ESC) guidelines [2], and fulfilled the criteria for an early invasive strategy, and underwent coronary angiography within 24 h after admission. Patients with NSTEMI and confirmed coronary artery disease (CAD) treated by PCI were involved in the analysis. Patients with CAD qualified for surgery revascularization, patients with MINOCA (myocardial infarction with nonobstructive coronary arteries), prior myocardial infarction, prior PCI, prior coronary artery bypass grafting (CABG), and patients treated noninvasively were excluded from the analysis. 

The patients were divided into two groups based on coronary angiography results: baseline Thrombolysis in Myocardial Infarction (TIMI) flow 0 of IRA (TO group); and baseline TIMI flow ≥ 1 of IRA (non-TO group). 

The following clinical factors were analyzed:

- demographic data: age, gender, BMI;
- co-morbidities, including systemic arterial hypertension, atrial fibrillation (AF), type 2 diabetes mellitus (DM), and peripheral occlusive artery disease (POAD);
- case history, involving data on current smoking, previous stroke, and family history of CAD;
- laboratory tests: baseline troponin, fasting glucose, creatinine, estimated glomerular filtration rate (eGFR), total cholesterol (TCh), serum triglycerides (TG), serum high-density lipoprotein (HDL), serum low-density lipoprotein (LDL);
- left ventricular ejection fraction (LVEF) measured by 2D transthoracic echocardiography at admission and discharge;
- Global Registry of Acute Coronary Events (GRACE) risk score [2];
- invasive coronary angiography with special focus on the presence of multivessel disease (MVD) and the culprit lesion location in the coronary arteries: left anterior descending artery (LAD), left main coronary artery (LM), right coronary artery (RCA), and left circumflex coronary artery (LCx);
- time to PCI (door-PCI) and final TIMI score (3 vs. non-3) after PCI.

### 2.2. Definitions Used in the Study

NSTEMI was defined according to the ESC fourth universal definition of myocardial infarction [6]. Diagnostic and treatment strategy was performed according to the 2015 NSTEMI ESC guidelines [2].

A coronary artery was considered an IRA (culprit) based on the following: ECG and angiographic features (definite or suspected thrombus, ruptured or ulcerated plaque, and the presence of TIMI grade flow ≤ 2). 

TIMI grade flow is a scoring system from 0–3 referring to levels of coronary blood flow assessed during angiography as follows: TIMI 0 flow (no perfusion); TIMI 1 flow (penetration without perfusion); TIMI 2 flow (partial reperfusion), and TIMI 3 is a normal flow that fills the distal coronary bed completely [9]. 

Body surface area (BSA) was estimated using the DuBois method [8]: BSA (m^2^) = (71.84 × weight (kg) 0.425 × height (cm) 0.725)/10,000. 

eGFR (mL/min/1.73 m^2^) was calculated by means of the Cockcroft–Gault formula normalized to 1.73 m^2^ ((140 × age in years) × weight (kg) × (0.85 if female) × 1.73 (m^2^))/(SCr(μmol/L) × 0.814 × BSA (m^2^)) [10,11]. 

### 2.3. Clinical Outcome

All subjects underwent a one-year follow-up. In-hospital and one-year mortality was analyzed.

### 2.4. Statistical Analysis

The study population was first dichotomized into two groups, occluded versus nonoccluded culprit artery. Clinical characteristics and outcomes were compared between groups. Continuous variables were presented as arithmetic mean and standard deviation (SD), and categorical as absolute values and percentages. Normality was verified using the Shapiro–Wilk test. The comparisons of groups were based on Student’s two-sample t-tests or nonparametric Mann–Whitney U tests as appropriate. Wilcoxon’s test was used for paired samples. The differences in proportions between groups were analyzed using the χ^2^ test. Univariate analysis was applied to both continuous and categorical variables. Stepwise multivariable logistic regression analyses were performed to establish the relationship between the patient characteristics and the presence of TO (variables with *p* < 0.10 in univariate model). A significance level of 0.05 was required for a variable to stay in the model, and for all of the used tests. All analyses were performed using Statistica 10.0 (StatSoft Polska, Kraków, Poland) software.

## 3. Results

### 3.1. Clinical Characteristics

A total of 399 patients were included in the study. The number of patients with preprocedural TIMI 0 (TO group) was 138, and the number with preprocedural TIMI 1–3 was 261. The patients with TO were more often smokers (*p* = 0.03) and had a greater incidence of diabetes mellitus (*p* = 0.003) and atrial fibrillation (*p* = 0.028) in comparison to non-TO patients. An additional factor positively affecting the occurrence of TO was a family history of CAD in relatives of the 1st degree (*p* < 0.001). The patients with initial TIMI 0 had higher baseline troponin level (*p* = 0.004), higher serum triglycerides (*p* = 0.001), and glucose level (*p* < 0.001). The LVEF values both at admission and discharge were significantly lower in TO than non-TO groups. GRACE risk score was similar in TO and non-TO groups (Table 1). 

### 3.2. Coronary Angiography

Coronary angiography confirmed CAD in all study subjects, and revealed TO in 138 (34.6%) patients. The left circumflex artery (LCx) was the most frequent IRA in the TO group (39.1% vs. 30.9% in the non-TO group, *p* = 0.001), whereas the left anterior descending artery (LAD) was more common in the non-TO group (48.5% vs. 27.1% in TO-group, *p* = 0.035). MVD was diagnosed in 62.3% patients with TO, and in 67.4% patients without TO of IRA. The time to PCI was comparable between the groups, and the final TIMI 3 flow score after PCI was registered more often in non-TO patients (*p* = 0.001) (Table 1).

### 3.3. Risk Factors for TO of IRA in NSTEMI Population

Univariate logistic regression analysis revealed that the following factors were associated with the incidence of TO of IRA: male gender (odds ratio (OR) 2.093, *p* = 0.004); atrial fibrillation (OR 1.781, *p* = 0.015); diabetes mellitus (OR 1.635, *p* = 0.019); serum triglycerides (OR 1.003, *p* = 0.046); family history of CAD (OR 2.790, *p* < 0.001); and HDL level (OR 0.972, *p* < 0.001). Multivariate analysis identified the following independent predictors for TO of IRA: LVEF (OR 0.949, *p* < 0.001); family history of CAD (OR 2.630, *p* < 0.001); and HDL level (OR 0.972, *p* = 0.002). Results of univariate logistic regression and multivariate analysis are presented in Table 2. 

### 3.4. Clinical Outcomes

In-hospital and one-year mortality were significantly higher in the TO group than in non-TO group (2.8% vs. 1.1%, *p* = 0.007 and 18.1% vs. 6.5%, *p* < 0.001, respectively). The independent predictors of in-hospital mortality were: LVEF at admission (OR 0.768, *p* = 0.004) and TO of IRA (OR 1.863, *p* = 0.005).

Independent predictors of one-year mortality were: LVEF at admission (OR 0.950, *p* < 0.001); hemoglobin level (OR 0.698, *p* < 0.001); pulmonary disease (OR 2.291, *p* = 0.033); age (OR 1.062, *p* = 0.003); thrombocytes level (OR 1.006, *p* = 0.008); and glucose level (OR 1.003, *p* = 0.047).

## 4. Discussion

Our results provide contemporary data on the incidence, predictors, and clinical significance of TO of IRA in patients with NSTEMI undergoing percutaneous coronary intervention. In this prespecified NSTEMI population, TO of IRA occurred in 34.6% of patients. The following factors: gender; atrial fibrillation; diabetes mellitus; family history of CAD; serum triglycerides; and HDL level were associated with the occurrence of TO of IRA. Finally, the independent predictors for TO were left ventricle ejection fraction, family history of CAD, and a low serum HDL level. Both in-hospital and one-year mortality were significantly higher in the TO group. TO of IRA was an independent predictor of in-hospital mortality, and, on the other hand, one-year mortality had a multifactorial origin. 

In our population, TO of IRA was revealed in 34.6% of patients. However, we should be aware that the NSTEMI group is very heterogeneous, which triggers diagnostic problems. The main NSTEMI population is characterized by nonoccluded IRAs, and those who only have subendocardial ischemia [12,13]. Another NSTEMI subgroup comprises patients with gradual progression of atheromatous plaque to TO, with well-developed collaterals preventing transmural ischemia and ST-segment elevation on ECG [14]. 

NSTEMI patients with TO of IRA were characterized by higher cardiovascular risk. They were more often current smokers, had a higher incidence of hypertension, DM, and AF. These results may slightly differ from those reported in the registries comparing STEMI and NSTEMI populations where patients with TO were younger, more often current smokers, and had a lower incidence of hypertension and diabetes mellitus [12]. 

It has been known that early reopening of a completely occluded coronary artery reduces myocardial damage, prevents heart failure, and improves clinical outcomes [15,16]. In our patients with TO of IRA, the troponins level was higher, and LVEF was lower than in non-TO patients. The research revealed statistically significant differences in in-hospital and one-year mortality in the TO subgroup, which is consistent with the findings of the Khan’s metanalysis [4]. The mentioned metanalysis suggests increased risk of the following major adverse cardiac events in the TO subgroup: death; non-fatal myocardial infarction; and target vessel revascularization. It seems important that in our study, the in-hospital mortality was associated with TO of IRA and LVEF at admission, but was not related to the standard risk score, such as the GRACE score. It should be stressed that one-year mortality was not related to TO of IRA but had a multifactorial origin. As we have mentioned, the NSTEMI patients with TO of IRA were characterized by higher cardiovascular risk and higher one-year mortality, probably increased by the co-morbidities.

In the literature, there is some data regarding TO of IRA in NSTEMI. A study conducted by Bahrmann et al. [17] showed that 29% of patients with NSTEMI were admitted with acute TO. This population was treated with an early invasive strategy with a higher rate of non-fatal reinfarction, with no difference in mortality rate compared to NSTEMI with non-TO. Wang’s research [18] highlighted that the culprit artery was occluded in 27% of NSTEMI patients, and this study group had larger infarct size, and higher short-term mortality, similar to our results. 

Our findings correspond, in many aspects, with preliminary data regarding predictive factors of TO of IRA. Aijaz et al. claimed that not only low LVEF was associated with TO of IRA, but also, higher age increased the occurrence of TO. [19] The meta-analysis by Hung CS and coworkers [20] showed that the overall estimated TO in NSTEMI was 34%, which conforms with our results. Furthermore, NSTEMI with TO was more frequently associated with LCx as the culprit artery, and lower LVEF, in agreement with our findings.

The Polish Registry of Acute Coronary Syndromes (PL-ACS including 2717 patients with NSTEMI in 2003–2006) showed TO of IRA in 26.6% of patients [21]. This is a slightly smaller percentage compared to our results. Both in our study and the cited research, the most frequent IRA with TO in the NSTEMI population was LCx (48.4% vs. 39.1%). On the other hand, our results revealed relatively high one-year mortality in TO, which was significantly higher than in the non-TO groups (18.1% vs. 6.5%, *p* < 0.001, respectively). In contrast to our results, Karwowski et al. [12] showed no differences in 12- and 36-month mortality in the TO and non-TO NSTEMI patients. Patients with TO in LCx had higher in-hospital mortality. Moreover, TO of Cx was associated with higher 36-month mortality in subjects with the delayed angioplasty, performed >2h after admission [13,22].

Considering the high practical importance of the topic, a variety of papers addressing biomarkers useful in early detection of TO in IRA in NSTEMI were published. They include novel biomarkers, i.e., dephosphorylated-uncarboxylated matrix gala protein (dp-ucMGP), the level of which was increased in patients with NSTEMI, and a higher GRACE score [23]. Interestingly, the highest tertile of plasma dp-ucMGP levels were found in patients with a clinical profile similar to the one recognized in our patients with TO in IRA in NSTEMI [23]. 

### Limitations of the Study

Because of the retrospective character, our study came up with several limitations. We have analyzed the NSTEMI population hospitalized in 2019 and managed it according to the 2015 ESC guidelines. Our 2020 experience has been influenced by the COVID-19 pandemic that limited the analysis, and, as of now, 2019 was the last period in which reasonable analysis could occur. The present study covered only NSTEMI subjects who were submitted to percutaneous coronary intervention, hence, our analysis did not involve patients referred for surgical revascularization, nor patients without coronary lesions suitable for intervention.

We are aware that some ECG markers may be helpful for risk assessment in NSTEMI subjects [24,25]. However, we have focused on the analysis of non-ECG clinical factors. There was no collection of accurate ECG data and information on the use of any additional leads. Our laboratory tests were limited to the essential markers—thus, we have no data regarding novel biomarkers suggesting TO. The culprit arteries were determined by one experienced invasive cardiologist in the catheterization laboratory (using electrocardiographic and angiographic findings) to limit the problem with selecting the IRAs among the patients with multivessel disease. Patients with previous myocardial infarctions, PCIs, and CABGs were excluded from the study, limiting errors in the culprit vessel interpretation in these subjects. We have no detailed data on the causes of death—thus, we have analyzed the total mortality only. 

## 5. Conclusions

The NSTEMI population is heterogeneous concerning both clinical presentation and angiographic findings. Despite that, the results of our study can be relevant for the population of NSTEMI patients with high-risk criteria in whom early invasive strategy is planned from the very beginning. According to the current guidelines [1], an early invasive strategy within 24 h is recommended in all NSTEMI patients. An immediate invasive angiography is recommended in unstable NSTEMI patients according to hemodynamic status, arrhythmias, acute heart failure, or persistent chest pain [1]. 

Non-invasive identification of TO of IRA in NSTEMI patients is still challenging. In patients with NSTEMI, TO of IRA represents a considerably frequent phenomenon, and corresponds with established clinical markers of impaired outcomes. These patients may require a different clinical approach than typical NSTEMI patients. Therefore, the utmost caution should be paid to prevent delay of coronary angiography in male NSTEMI patients with impaired left ventricular systolic function, metabolic disturbances, and a family history of CAD, who share the increased risk of acute TO. Our results suggest that in patients with a high risk of TO of IRA coronary occlusion, an immediate, but not-early, invasive strategy is a reasonable option to salvage the myocardium and improve prognosis.

## Figures and Tables

**Table 1:** Baseline characteristics and risk factors of TO of IRA.

		TO Group (*n* = 138)	Non-TO Group (*n* = 261)	*p*-Value
Demographic Data:				
Age (years)		71 ± 10.1	71 ± 10.1	0.670
Male gender		113 (81.9%)	180 (70.0%)	0.005
BMI (kg/m^2^)		29.3 ± 5.2	28.1 ± 4.5	0.160
Concomitant diseases:				
Arterial hypertension		120 (87%)	212 (81.2%)	0.160
AF		44 (31.9%)	57 (21.8%)	0.028
Diabetes mellitus type 2		70 (50.7%)	92 (35.2%)	0.003
POAD		30 (21.7%)	47 (18%)	0.370
Case medical history:				
Current smoking		47 (34.1%)	67 (25.7%)	0.030
Previous stroke		11 (8%)	25 (9.6%)	0.600
Pulmonary disease		28 (20.3%)	40 (15.3%)	0.030
Family history CAD		50 (36.2%)	51 (19.5%)	<0.001
Laboratory tests:				
Baseline troponin (μg/L)		0.067 ± 0.71	0.042 ± 0.65	0.004
Glucose (mg/dL)		123 ± 81.6	110 ± 75.4	<0.001
Hemoglobin (g/dL)		14.66 ± 10.5	13.78 ± 8.37	<0.001
Thrombocytes level (×1000/mm^3^)		223.17 ± 70.87	217.02 ± 70.05	0.008
Creatinine (mg/dL)		1.045 ± 12.8	1.02 ± 11.2	0.500
eGFR (mL/min)		70 ± 28.7	75 ± 23.5	0.020
TCh (mg/dL)		152 ± 49	150 ± 36	0.740
TG (mg/dL)		129 ± 73	109 ± 65	0.001
HDL (mg/dL)		40 ± 14.3	43 ± 12.7	0.090
LDL (mg/dL)		82 ± 35.1	77 ± 31.1	0.300
Echocardiography:				
LVEF at admission (%)		39.73 ± 13.10	48.05 ± 11.71	<0.001
LVEF at discharge (%)		37.94 ± 14.06	46.60 ± 12.04	0.001
GRACE score (points)		139.6 ± 22	132.5 ± 26	0.341
Invasive coronary angiography:				
MVD		86 (62.3%)	176 (67.4%)	0.081
Culprit Lesion	LAD	38 (27.1%)	127 (48.5%)	0.035
	LCx	53 (39.1%)	81 (30.9%)	0.001
	RCA	45 (32.5%)	47 (18.2%)	0.080
	LM	2 (1.3%)	6 (2.4%)	0.510
Time to PCI (hour)		3.62 ± 0.7	3.96 ± 0.9	0.238
Final TIMI 3 flow		110 (79.7%)	252 (96.6%)	0.001

Data are presented as number and percentage (in brackets) of patients, or mean ± standard deviation; TO—total occlusion; non-TO—non-total occlusion; BMI—body mass index; AF—atrial fibrillation; POAD—peripheral occlusive artery disease; CAD—coronary artery disease; eGFR—estimated glomerular filtration rate; TCh—total cholesterol; TG—triglycerides; HDL—high density lipoprotein; LDL—low density lipoprotein; LVEF—left ventricle ejection fraction; GRACE—Global Registry of Acute Coronary Events; MVD—multivessel disease; LAD—left anterior descending artery; LCx—left circumflex coronary artery; RCA—right coronary artery; LM—left main coronary artery; PCI—percutaneous coronary intervention; TIMI—thrombolysis in myocardial infarction.

**Table 2:** Univariate analysis and logistic regression—the risk factors for TO of IRA.

		Univariate Analysis			Logistic Regression		
		OR	95% CI	*p*	logOR	95% CI	*p*
Demographic data:							
Age (years)		0.999	0.979–1.020	0.994			
Male		2.093	1.262–3.472	0.004			
BMI (kg/m^2^)		1.027	0.976–1.079	0.309			
Concomitant diseases:							
Arterial hypertension		0.994	0.537–1.839	0.985			
AF		1.781	1.116–2.843	0.015			
Diabetes mellitus		1.635	1.084–2.466	0.019			
POAD		1.250	0.753–2.076	0.388			
Case medical history:							
Current smoking		1.304	0.839–2.027	0.238			
Pulmonary diseases		2.291	1.071–4.898	0.033			
Previous stroke		0.919	0.432–1.954	0.826			
Family history CAD		2.790	1.734–4.492	<0.0001	2.630	1.567–4.415	<0.001
Laboratory tests:							
Baseline troponin (μg/L)		1.324	0.963–1.821	0.084			
Glucose (mg/dL)		1.003	0.999–1.006	0.078			
Creatinine (mg/dL)		0.980	0.904–1.062	0.621			
GFR (mL/min)		0.997	0.986–1.007	0.508			
TCh (mg/dL)		0.998	0.993–1.004	0.502			
TG (mg/dL)		1.003	1.000–1.006	0.046			
HDL (mg/dL)		0.972	0.956–0.988	0.001	0.972	0.955–0.989	0.002
LDL (mg/dL)		1.002	0.995–1.008	0.613			
Hemoglobin level (g/dL)		0.698	0.576–0.847	0.001			
Thrombocytes level (×1000/mm^3^)		1.006	1.002–1.011	0.008			
Echocardiography:							
LVEF at admission (%)		0.949	0.9328–0.966	<0.0001	0.950	0.931–0.968	<0.0001
LVEF at discharge (%)		0.951	0.9243–0.978	0.001			
GRACE score		1.039	0.978–1.056	0.067			
Invasive coronary angiography:							
MVD		1.006	0.997–1.028	0.601			
Culprit Lesion	LAD	1.137	0.6996–1.847	0.605			
	LCx	0.832	0.5110–1.354	0.458			
	RCA	1.255	0.7758–2.0295	0.355			
	LM	-	-	-			

TO—total occlusion; non-TO—non-total occlusion; BMI—body mass index; AF—atrial fibrillation; POAD—peripheral occlusive artery disease; CAD—coronary artery disease; GFR—glomerular filtration rate; TCh—total cholesterol; TG—triglycerides; HDL—high density lipoprotein; LDL—low density lipoprotein; LVEF—left ventricle ejection fraction; GRACE—Global Registry of Acute Coronary Events; MVD—multivessel disease; LAD—left anterior descending artery; LCx—left circumflex coronary artery; RCA—right coronary artery; LM—left main coronary artery; PCI—percutaneous coronary intervention; TIMI—thrombolysis in myocardial infarction.
